# Relationships Among Dietary Cognitive Restraint, Food Preferences, and Reaction Times

**DOI:** 10.3389/fpsyg.2019.02256

**Published:** 2019-10-09

**Authors:** Travis D. Masterson, John Brand, Michael R. Lowe, Stephen A. Metcalf, Ian W. Eisenberg, Jennifer A. Emond, Diane Gilbert-Diamond, Lisa A. Marsch

**Affiliations:** ^1^Norris Cotton Cancer Center, Department of Epidemiology, Geisel School of Medicine at Dartmouth College, Hanover, NH, United States; ^2^Department of Psychology, Drexel University, Philadelphia, PA, United States; ^3^Center for Technology and Behavioral Health, Geisel School of Medicine at Dartmouth College, Lebanon, NH, United States; ^4^Department of Psychology, Stanford University, Stanford, CA, United States; ^5^Department of Biomedical Data Sciences, Geisel School of Medicine, Dartmouth College, Hanover, NH, United States

**Keywords:** Three-Factor Eating Questionnaire, TFEQ, dietary cognitive restraint, food preference, reaction time

## Abstract

**Objective:**

To assess the associations between dietary cognitive restraint, disinhibited eating, and how taste and health perceptions relate to food preference; and further, whether cognitive restraint and disinhibited eating are associated with food preference decision reaction time.

**Methods:**

Five hundred and seventeen adults participated in the study. Dietary cognitive restraint and disinhibited eating were assessed using the shortened Three-Factor Eating Questionnaire (TFEQ-R18). Participants also completed a dietary decision-making task to examine their food-related decisions. Participants were presented with 50 food items and asked to rate them for health and for taste. Participants were then presented with a reference food item and comparison items one at a time and asked to indicate which of the two foods they would prefer to eat.

**Results:**

Participants with higher levels of cognitive restraint were more sensitive to health perceptions whereas those with higher levels of disinhibited eating were more sensitive to taste perceptions when indicating food preference. Reaction time analysis corroborated these results. Being classified as high for cognitive restraint was associated with faster reaction times if the preferred food was rated as healthier than the referent food. Conversely, being classified as high for disinhibited eating was associated with faster reaction times if the preferred food was rated as tastier than the referent food.

**Conclusion:**

The dietary decision-making task appears to capture distinct aspects of dietary restraint and disinhibition and may be useful in future studies to measure and/or alter levels of dietary restraint and disinhibition.

## Introduction

Obesity has reached pandemic levels worldwide (Ng et al., 2014). One of the most common strategies employed by individuals attempting to reduce weight is modifying their dietary intake to consume fewer calories (Kruger et al., 2004). To accomplish this, these individuals must often place themselves in a state of dietary cognitive restraint to control their eating behavior (Lowe and Kleifield, 1988). An individual’s level of dietary cognitive restraint [the intent to reduce their energy intake (Keränen et al., 2009)] or disinhibited eating [the tendency to eat in response to social and emotional cues or the availability of palatable foods in the environment (Stunkard and Messick, 1985)] has been shown to have a statistically significant relationship to energy intake and weight. For example, individuals with a combination of high dietary cognitive restraint and low disinhibited eating are more successful at maintaining weight loss (Keränen et al., 2009).

Supporting these findings, other studies have reported that individuals with higher levels of reported dietary cognitive restraint are able to maintain weight loss (Foster et al., 1998; Sarlio-Lähteenkorva and Rissanen, 1998; Westerterp-Plantenga et al., 1998; Drapeau et al., 2003; Chaput et al., 2007; Vogels and Westerterp-Plantenga, 2007) whereas those with higher disinhibited eating scores tend to have higher body weights (Lindroos et al., 1997; Fogelholm et al., 1999; Provencher et al., 2003; Dykes et al., 2004). Dietary cognitive restraint and disinhibition have also been shown to predict self-reported energy intake. This dovetails with the finding that dietary cognitive restraint has also been positively associated with self-reported consumption of healthy foods (de Lauzon et al., 2004; Keränen et al., 2011; Spence et al., 2016). However, it has been suggested that restrained eaters eat no less in the natural environment than unrestrained eaters (Stice et al., 2004). It has alternatively been argued that these real-world observations mean that restrained eaters are eating less than they would like to eat but not less than they need to eat to reduce their weight (Lowe and Levine, 2005).

The shortened version of the Three-Factor Eating Questionnaire (TFEQ-R18) (Keränen et al., 2009) has been extensively used to describe eating behaviors in both clinical and general populations. In general, this scale measures an individual’s level of dietary cognitive restraint and disinhibited eating. For example, the TFEQ-R18 contains a subscale to measure an individual’s level of dietary cognitive restraint (CR), and subscales to measure emotional eating (EE), and uncontrolled eating (UE), both of which are thought to reflect an individual’s level of disinhibited eating (Pentikäinen et al., 2018). However, much of the work done with the TFEQ-R18 relies on self-reported measures of diet and food choice.

It may be beneficial to examine the relationship between dietary measures of restraint and disinhibition in relation to food choices in real time rather than by self-report to allow for the examination of contemporaneous decision making rather than relying on long term memory. For example, reporting the frequency of foods eaten throughout the year is a process distinct from making momentary decisions about which foods you would prefer to eat. The moment to moment decisions are likely to be more important to those who are attempting to control their dietary energy intake. Dietary decision-making tasks allow for this type of evaluation (Hare et al., 2009).

In a dietary decision-making task, participants first rate a variety of foods on measures of healthiness and tastiness. Participants then complete a series of trials where they are asked to report their preference for various foods. Combining a participant’s health and taste ratings with their food preferences can provide insight into how the individual’s decisions are influenced by their perception of a food’s healthiness or tastiness. Because taste and health sensitivity may be quickly evaluated in real time using these tasks, alterations to these metrics from an intervention could be rapidly observed. This would reduce the need for self-reporting which is both burdensome and time consuming for participants and researchers. This task is also easy to administer and can be administered multiple times throughout a week or even a day to assess both short- and long-term temporal changes in dietary preferences. Further, as the stimuli in the dietary decision-making task can be easily modified, there is great flexibility for training paradigms or for the study of specific eating behavior constructs, such as the study of cravings.

Additionally, the time it takes for a participant to indicate a preferred food can be measured as reaction times during a dietary decision-making task. Research suggests that it takes longer to process abstract attributes like a food’s health compared to basic attributes like a food’s taste (Sullivan et al., 2014). For example, Sullivan et al. (2014) used reaction time in a dietary choice task to show that tastiness was processed approximately 190 ms faster than the healthiness of a food. Because individuals with higher levels of cognitive restraint may have greater consideration of the healthiness of foods when selecting between two food options, they may take longer to choose a food than individuals with lower cognitive restraint. In contrast, those with high disinhibition may be primarily driven by the tastiness of foods and so reaction times may be lower than those with low disinhibition.

Reaction times may also serve as an implicit cognitive measure that may be used to investigate the level of conflict experienced by people making food decisions. For example, a study of weight-concerned women reported that reaction times were higher when participants selected the healthy option between two foods conflicted by health and taste (e.g., choosing between a healthy and hedonic food) (van der Laan et al., 2014). Building on this finding, it is possible that reaction times may increase when the choice between foods is less distinct in terms of health and/or taste. Additionally, compared to participants with low levels of trait self-control, those with high levels have been shown to have less conflict between healthy and unhealthy food items, as measured by their subjective positive and negative evaluations of pictured foods (Gillebaart et al., 2016). In the same study, higher trait self−control was associated with faster resolution of conflict, as indexed by reaction time, in a categorization task that required participants to categorize healthy and unhealthy food images as either positive or negative. For example, compared to participants with high self-control, participants with low self-control took longer to categorize unhealthy foods as negative. Because the TFEQ is a measure of dietary self-control, reaction times of food choices may therefore be influenced by levels of restraint or disinhibition although this is not well-examined in the literature. It is therefore beneficial to interrogate whether levels of cognitive restraint and disinhibited eating are related to reaction times during dietary decision-making tasks. The presence of distinct relationships between cognitive restraint and reaction times and between disinhibited eating and reaction times would provide an internal cognitive validation of the use of such tasks to distinguish between a person’s restraint and disinhibition.

The purpose of the present work is to assess the relationship among cognitive restraint, disinhibited eating, and outcomes from a dietary decision-making task, including participant health sensitivity, taste sensitivity, proportion of healthy options and tasty options preferred, as well as food preference reaction times. To accomplish this, we leveraged an existing data set of survey responses collected from a larger project designed to model the ontological structure of self-regulation in 522 participants (Eisenberg et al., 2018, 2019). As part of a battery of online tasks and questionnaires presented using the Experiment Factory framework (Sochat et al., 2016) through Amazon Mechanical Turk (MTurk), participants completed both the dietary decision-making task and the TFEQ-R18. We hypothesized that dietary cognitive restraint, as measured by the TFEQ-R18, is positively related to a participants’ health sensitivity and the proportion of healthy options preferred while not being related to taste sensitivity and the proportion of tasty options preferred. We hypothesized that the opposite is true for disinhibited eating. As some previous work has suggested that taste information is processed faster than health information (Sullivan et al., 2014) we hypothesized that those with higher levels of dietary cognitive restraint would be slower in indicating preference for a food if the preferred food is healthier or if the preferred food is rated higher in health than taste. Conversely, those reporting higher levels of disinhibited eating behaviors may place more emphasis on taste and therefore may have faster reaction times for indicating preference when a preferred food is tastier or when the preferred food is rated higher in taste than health.

## Materials and Methods

### Participants

Survey responses were collected from a larger project designed to model the ontological structure of self-regulation in 522 participants (Sochat et al., 2016; Eisenberg et al., 2018, 2019). Each participant provided informed consent and received monetary compensation for participating in the study. All study protocols were approved by Dartmouth’s Committee for the Protection of Human Subjects and Stanford’s Institutional Review Board. For transparency purposes, the analysis plan for this secondary analysis was pre-registered on an Open Science Framework page available here: https://osf.io/um2r7/. Similarly, all data and analysis scripts are available on the same page. The original study was advertised on MTurk. Participants were informed about the original study’s aims and hypotheses; however, as the study reported here is a secondary analysis, participant were not aware that such an analysis would be conducted. The inclusion criteria for the original study were adults between 18 and 50 that resided within the United States of America. For completion of the total battery of questions, participants were paid $60 (Eisenberg et al., 2018, 2019).

### Demographic Variables

As part of the larger survey, participants self-reported a variety of demographic variables including age, sex, height, weight, race, highest household income, and education. Body mass index (BMI) values were calculated from height and weight by dividing weight in kg by height in meters squared (kg/m^2^). Participants were grouped into weight status categories using standard Centers for Disease Control and Prevention (CDC) BMI cut points (healthy weight: ≤24.9, overweight: 25–29.9, obese: ≥30) (CDC, 1998).

### Eating Behavior Measures

Participants completed the TFEQ-R18. The TFEQ-R18 is a validated (Keränen et al., 2009, 2011) and shortened version of the original Three-Factor Eating Questionnaire (TFEQ) (Stunkard and Messick, 1985). The TFEQ-R18 was originally intended for use in populations with obesity and has been validated for use within the general population (Pentikäinen et al., 2018). The TFEQ-R18 measures both behavioral and cognitive components of eating behavior using three subscales: cognitive restraint (CR), uncontrolled eating (UE), and emotional eating (EE). The cognitive restraint subscale contains six items that are thought to measure an individual’s tendency to restrict dietary intake to control weight (e.g., “I deliberately take small helpings as a means of controlling my weight”). The uncontrolled eating subscale includes nine items that are thought to measure an individual’s control over their eating behavior (e.g., “Sometimes when I start eating, I just can’t seem to stop”). The emotional eating subscale includes three items and is thought to measure an individual’s tendency to eat in response to negative emotions (e.g., “When I feel blue, I often overeat”). TFEQ-R18 subscale scores were converted to a scale ranging from 0 to 100 using the following equation: [(raw score - lowest possible raw score)/possible raw score range) × 100]. Therefore, higher scores indicate greater tendencies toward dietary cognitive restraint (CR subscale), losing control over eating behavior (UE subscale), or greater likelihood of eating in response to negative emotions (EE subscale).

The internal-consistency of subscales was estimated using Cronbach’s alpha for each scale (cognitive restraint = 0.84, uncontrolled eating = 0.88, emotional eating = 0.90). In an initial overview of our data we found that uncontrolled eating and emotional eating were highly correlated (*r* = 0.70, *p* < 0.001). Because of this and because both measures are thought to be indicators of general disinhibited eating (Stunkard and Messick, 1985; Karlsson et al., 2000; Pentikäinen et al., 2018) we created a mean disinhibited eating score by averaging uncontrolled eating and emotional eating scores for each participant. This disinhibited eating score was used in our analyses to reduce multiple comparisons and make more straightforward inferences in regard to disinhibition.

### Dietary Decision-Making Task

The dietary decision-making task has been used previously and is designed to examine participants’ food-related decisions in relation to both perceived taste and perceived health of a variety of food items (Hare et al., 2009). The stimuli from the original Hare et al. (2009) experiment were used for this study. Stimuli consisted of pictures of a variety of foods (e.g., cookies, chocolate bars, yogurt, strawberries, celery, etc.); a complete list of included food items can be found in Supplementary Table 1. All food items were displayed on a black background during the task. A demonstration of the experiment including the underlying code and stimuli can be found here: https://expfactory-experiments.github.io/dietary-decision/. The task consists of two phases. In the first phase, participants rate both the tastiness and healthiness of 50 distinct food items on a five-point Likert scale. Ratings were anchored on five-point scales; taste ratings were anchored from 1 = “very bad” to 5 = “very good” and health ratings were anchored at 1 = “very unhealthy” to 5 = “very healthy.” From these 50 foods, a food item that falls closest to the median on both taste and health is selected as a referent item. In the second phase, participants are presented with the referent food item and all remaining 49 items (target items) one at a time on individual trials. Participants are asked to indicate on each trial whether they would prefer the target food over the referent food on a five-point Likert scale ranging from a “Strong No” indicating preference for the referent item to a “Strong Yes” indicating preference for the target food. The Likert point centered in the middle represents neutral, indicating no preference for either the target or referent food. Reaction time to indicate food preference on each trial was calculated by subtracting the time at which the participant responded from the time of stimuli onset. Responses were made via keyboard and reaction times were not speeded. Reaction times were measured to the nearest millisecond.

### Health Sensitivity and Taste Sensitivity Scores

Because participants rate a variety of foods on both health and taste, it is possible to estimate how much their health ratings and taste ratings are associated with their food preferences. For example, if a participant indicates preference for a food that is high in taste but low in health compared to a food moderate in taste but high in health, it is possible to infer that taste played a larger role in their preference. Health sensitivity may be defined as the association between a participant’s health ratings and their food preference and taste sensitivity may be defined as the association between a participant’s taste ratings and their food preference. These scores are calculated by computing a linear regression model for each participant. Food preference ratings for the target compared to the referent food ranging from −2 (strong no preference for the target food) to 2 (strong yes preference for the target food) are predicted from the difference between the health and taste scores for the target food compared to the referent food on each trial. The model thus yields two coefficient values for each individual, approximating how much taste (taste sensitivity) and health (health sensitivity) related to the food preferences indicated during the dietary decision-making task.

### Health Difference, Taste Difference, and Health-Taste Difference Scores

Three hybrid variables were created to index the difference between the health and the taste ratings between the preferred foods and the non-preferred foods. All variables were calculated on a trial level basis, for trials on which a food preference was indicated. The first was a health difference score that compared the health rating of the preferred food to that of the non-preferred food. Health difference scores were computed for each trial by subtracting the un-preferred food’s health rating from the preferred food’s health rating. Values are ordinal ranging from −8 to 8. A positive value indicates that the preferred food had a higher health rating than the non-preferred food, with higher values indicating greater perceived healthiness for the preferred food. Conversely, a negative value indicates that the non-preferred food had a higher health rating than the preferred food, with lower values indicating greater perceived health for the non-preferred food. An analogous taste difference score was created that compared the taste rating of the preferred food to the taste rating of the non-preferred food.

We also created a health-taste difference score to indicate whether the preferred food had a higher health or taste rating normalized to the non-preferred food. The score was calculated by subtracting the taste difference score from the health difference score described above. A score of zero indicates that the difference between the preferred food and the non-preferred food in terms of healthiness and tastiness ratings were equal; a negative value indicates that the preferred food was tastier than it was healthier in comparison to the non-preferred food; and a positive value indicates that the preferred food was healthier than it was tastier in comparison to the non-preferred food. The calculation of this score provided values running from −8 to 8. An inspection of the distribution revealed that number of observations at the extremes (< −5 and >5) did not occur often (< 50 per cell compared to >1000 per cell at the non-extremes) resulting in poor precision when calculating point estimates. To improve the estimation precision values of −8, −7, and −6 were recoded as −5 and values of 6, 7, and 8 were recoded as 5. This resulted in a final range of scores from −5 to 5. However, analyses were run both with and without the collapsed values with similar results. Non-collapsed results are displayed in Supplementary Table 2.

### Proportion of Healthy Options Preferred and Proportion of Tasty Options Preferred

The proportion of healthy and the proportion of tasty options preferred were calculated for each participant. The proportion of healthy options was calculated by dividing the number of trials on which the health difference score was greater than zero by all trials completed. The proportion of tasty options preferred was calculated by dividing the number of trials on which the taste difference score was greater than zero from by all trials completed.

### Statistical Analysis

All analyses were conducted in R-Studio (v1.1.463). Measures were first inspected for plausibility and normality. There were three individuals who reported being less than 2 feet tall and one individual who had a calculated BMI of 3; all four individuals were removed from the analyses. One additional individual was excluded for indicating the same response to all questions during the dietary decision-making task. Prior to all reaction time analyses, a reaction time outlier analysis was conducted to remove reaction time trials if they were ±2.2 standard deviations from the mean (Hoaglin et al., 1986; Hoaglin and Iglewicz, 1987). Additionally, because previous studies have observed differences between levels of dietary cognitive restraint and disinhibited eating (Price et al., 2015) by weight status, both demographic and TFEQ-18 scores were stratified and appropriate tests (ANOVA or Chi-square) were conducted to assess differences between groups.

To determine potential covariates, pair-wise correlations were calculated between demographic variables of interest (i.e., age, sex, BMI) and all outcome variables including: taste sensitivity, health sensitivity, proportion of healthy options preferred, proportion of tasty options preferred, dietary cognitive restraint scores, uncontrolled eating scores, emotional eating scores, and reaction times. Any demographic variables which statistically significantly correlated (*p* < 0.10) with any outcome variables were included as covariates in later analyses.

#### Associations Between Eating Behaviors and the Dietary Decision-Making Task Outcomes

Unadjusted linear regressions were used to fit outcomes from the dietary decision-making task (i.e., taste sensitivity, health sensitivity, proportion of healthy options chosen, proportion of tasty options chosen) on eating behaviors (i.e., dietary cognitive restraint and disinhibited eating). We then examined the adjusted associations between eating behaviors and outcomes from the dietary decision-making task using multivariable linear regression, adjusting for all relevant covariates. Because some predictor variables were on very different scales, all variables were standardized prior to analyses to enable equal comparisons of effect sizes.

#### Reaction Time Analysis

Unadjusted linear regressions were used to fit health score differences, taste score differences, dietary cognitive restraint, and disinhibited eating on reaction times. We then repeated the analyses to adjust for covariates. A sensitivity analysis was then computed by fitting health and taste differences on eating behavior outcomes simultaneously.

To address whether the level of dietary cognitive restraint modified the association between reaction time and health difference or the association between reaction time and taste difference, we included an interaction term between cognitive restraint and health ratings, or cognitive restraint and taste ratings, respectively; *p* < 0.10 based on the Wald’s *t*-test was considered evidence of a statistically significant interaction. A similar analysis was completed to assess whether disinhibited eating modified the effect of health rating or taste rating on reaction times. As a sensitivity analysis, we repeated our test for interactions using dietary cognitive restraint or disinhibited eating on a continuous scale.

We conducted a similar test for effect modification of dietary cognitive restraint and disinhibited eating within the health-taste difference measure. We estimated the effect of health-taste differences on reaction times, stratified by median dietary cognitive restraint and median disinhibited eating separately. The health-taste difference by median dietary cognitive restraint interaction and the health-taste difference by median disinhibited eating interaction were tested in separate models. As a sensitivity analysis, we repeated our test for interactions using dietary cognitive restraint or disinhibited eating as continuous variables.

## Results

### Participants and Covariates

Participant characteristics are summarized in Table 1. Of note, the level of dietary cognitive restraint was highest in those with obesity (M ± SD = 47.33 ± 20.88; *p* = 0.003) while uncontrolled eating and emotional eating scores were higher in those with overweight (uncontrolled eating: M ± SD = 44.22 ± 19.57, *p* < 0.001; emotional eating: M ± SD = 50.87 ± 28.34, *p* < 0.001). Significant Pearson’s correlations were observed between sex, age, race, BMI, and household income and at least one outcome variable of interest and were therefore included as covariates in subsequent analyses (see Supplementary Table 3 for complete results).

**TABLE 1:** Participant demographics by weight status.

	**Healthy weight**	**Overweight**	**Obese**	
	**(*n* = 218)**	**(*n* = 147)**	**(*n* = 152)**	
				
	***n* (%)**	***n* (%)**	***n* (%)**	***p*-value**
Males	112 (51.4)	83 (56.5)	64 (42.1)	**0.04**
**Race**				
White	177 (81.2)	127 (86.4)	128 (84.2)	
African American	10 (4.6)	13 (8.8)	10 (6.5)	
Other	31 (14.2)	7 (4.8)	14 (9.2)	**0.03**

	**Mean (±SD)**	**Mean (±SD)**	**Mean (±SD)**	

Age (years)	32.38 (7.49)	33.56 (7.88)	35.62 (8.14)	**<0.001**
Household	47413 (32099)	47319 (30148)	50189 (31435)	0.65
Income (USD)				
**TFEQ-R18 Subscale Scores**				
Dietary cognitive	39.58 (23.23)	41.46 (21.33)	47.33 (20.88)	**0.003**
restraint				
Uncontrolled eating	30.67 (18.62)	44.22 (19.57)	34.92 (18.35)	**<0.001**
Emotional eating	24.92 (25.40)	50.87 (28.34)	37.50 (29.10)	**<0.001**

**F*-values derived from one-way ANOVA. Bold values indicate the significance level is *p* < 0.05.*

### Associations Between Eating Behaviors and the Dietary Decision-Making Task

In the unadjusted models, as dietary cognitive restraint increased there was an associated increase in participants’ levels of health sensitivity (β = 0.16, *p* < 0.001) and the proportion of healthy options they preferred (β = 0.15, *p* < 0.001) (Table 2). As dietary cognitive restraint increased there was also a decrease in participants’ levels of taste sensitivity (β = −0.11, *p* = 0.02) and the proportion of tasty options they preferred (β = −0.10, *p* = 0.03). Additionally, as disinhibited eating scores increased, health sensitivity scores and the proportion of healthy options preferred decreased (β = −0.13, *p* = 0.004; *B* = −0.09, *p* = 0.05). Relationships were similar in the adjusted models for cognitive restraint and disinhibited eating, although the relationships associated with disinhibited eating were no longer statistically significant.

**TABLE 2:** Summary of the three linear regressions between standardized TFEQ-R18 and food decision-making task outcomes.

							**Proportion**					
	**Taste sensitivity**			**Health sensitivity**			**healthy preferred**			**Proportion tasty preferred**		
				
	***B***	**95% CI**	***p***	***B***	**95% CI**	***p***	***B***	**95% CI**	***p***	***B***	**95% CI**	***p***
**Unadjusted models^∗^**												
Cognitive restraint	**−0.11**	**(−0.19, −0.02)**	**0.02**	**0.16**	**(0.08, 0.25)**	**<0.001**	**0.15**	**(0.06, 0.24)**	**<0.001**	**−0.12**	**(−0.21, −0.04)**	**0.01**
Disinhibited eating	−0.03	(−0.12, 0.06)	0.49	**−0.13**	**(−0.21, −0.04)**	**0.004**	−0.09	(−0.17, −0.001)	0.05	−0.07	(−0.16, 0.02)	0.12
**Adjusted model^∗∗^**												
Cognitive restraint	**−0.13**	**(−0.22, −0.44)**	**0.003**	**0.15**	**(0.06, 0.24)**	**<0.001**	**0.15**	**(0.06, 0.24)**	**<0.001**	**−0.14**	**(−0.23, −0.05)**	**0.002**
Disinhibited eating	−0.03	(−0.12, −0.06)	0.53	−0.08	(−0.17, 0.01)	0.09	−0.08	(−0.17, 0.02)	0.10	−0.09	(−0.18, 0.004)	0.06

*^∗^Two separate linear regressions were run, one for dietary cognitive restraint and one for disinhibited eating. ^∗∗^Linear regressions were adjusted for age, sex, BMI, household income, and race. Both dietary cognitive restraint and disinhibited eating were included in this model together. Taste sensitivity, health sensitivity, proportion of healthy options preferred, proportion of tasty options preferred determined by the dietary decision-making task (Hare et al., 2009). Bold values indicate the significance level is *p* < 0.05.*

### Reaction Time Analysis

Reaction time was approximately 13 ms faster (β = −13.47, *p* < 0.001) with each 1 unit increase in the health difference between the preferred food and non-preferred food (Table 3). Similarly, for each 1 unit increase in the taste difference (ranging from −4 to 4) between the preferred food and non-preferred food individual’s reaction times were approximately 12 ms faster (β = −11.67, *p* < 0.001). Results were similar in both adjusted and unadjusted models. Dietary cognitive restraint and disinhibited eating were not associated with reaction times in either the unadjusted or adjusted models.

**TABLE 3:** Unadjusted and adjusted associations between reaction times (ms) and standardized cognitive restraint, disinhibited eating, and health and taste difference scores from the dietary decision-making task.

	**Unadjusted associations**			**Adjusted model^∗^**		
		
	***B***	**95% CI**	***p*-value**	***B***	**95% CI**	***p*-value**
Health difference	**−12.93**	**(−16.16, −9.71)**	**<0.001**	**−13.47**	**(−16.70, −10.24)**	**<0.001**
Taste difference	**−10.41**	**(−15.39, −5.44)**	**<0.001**	**−11.67**	**(−16.65, −6.69)**	**<0.001**
Cognitive restraint	0.38	(−0.74, 1.50)	0.51	0.20	(−0.90, 1.29)	0.73
Disinhibited eating	−0.25	(−1.36, 0.85)	0.65	0.20	(−0.94, 1.35)	0.73

*^∗^From a linear regression model with health difference and taste difference as the predictors and reaction time as the outcome, adjusted for age, sex, BMI, income, and race. Health and taste difference scores were calculated by subtracting the health and taste scores of the non-preferred food item from the preferred food item during the dietary decision-making task (Hare et al., 2009) ranged from −4 to 4. Dietary cognitive restraint and disinhibited eating values were calculated from the TFEQ-R18. Values ranged from 0 to 100. Bold values indicate the significance level is *p* < 0.05.*

**FIGURE 1:**
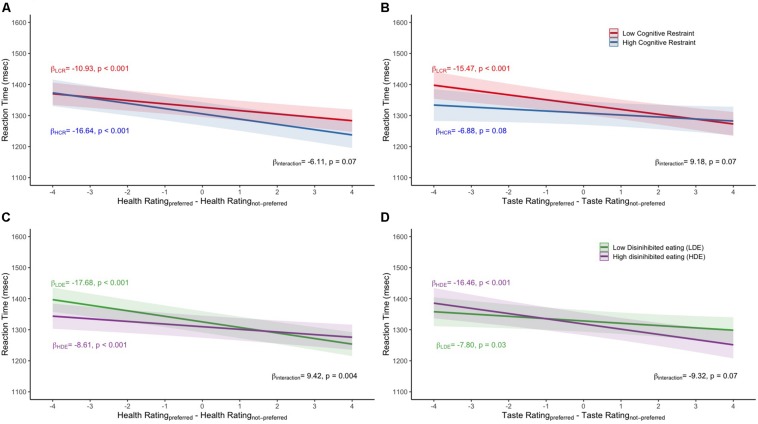
Mean reaction time values for the **(A)** median dietary cognitive restraint by health difference interaction, **(B)** median dietary cognitive restraint by taste difference interaction, **(C)** median disinhibited eating by health difference interaction, **(D)** median disinhibited eating by taste difference interaction. All models adjusted for age, sex, household income, and BMI. LCR, low dietary cognitive restraint; HCR, high dietary cognitive restraint; LDE, low disinhibited eating; HDE, high disinhibited eating.

There was a statistically significant interaction between median dietary cognitive restraint and health difference on reaction time (Figure 1A). Individuals with dietary cognitive restraint above the median were faster to indicate preferences (β = −16.64, *p* < 0.001) compared to those with dietary cognitive restraint below the median (β = −10.93, *p* < 0.001) when the preferred food was rated as healthier (*p* for interaction = 0.07). There was also a statistically significant interaction between median dietary cognitive restraint and taste difference (Figure 1B). Individuals with dietary cognitive restraint below the median were faster to indicate preference (β = −15.47, *p* < 0.001) compared to those with dietary cognitive restraint above the median (β = −6.88, *p* = 0.08) when the preferred food was rated as tastier (*p* for interaction = 0.07).

There was a statistically significant interaction between median disinhibited eating and health difference (Figure 1C). Individuals with disinhibited eating scores below the median were faster to indicate preference (β = −17.68, *p* < 0.001) compared to those above the median (β = −8.61, *p* < 0.001) when the preferred food was rated as healthier. There was also a statistically significant interaction between median disinhibited eating and taste difference (Figure 1D). Individuals with disinhibited eating scores above the median were faster to indicate preference (β = −16.46, *p* < 0.001) compared to those below the median (β = −7.80, *p* = 0.03) when the preferred food was rated as tastier. In the sensitivity analyses that treated dietary cognitive restraint as a continuous variable, the results corroborated the stratified analysis with a statistically significant interaction between dietary cognitive restraint and health difference predicting reaction time (*p* for interaction = 0.03). However, the taste difference and dietary cognitive restraint interaction predicting reaction time was not statistically significant (β = −0.11, *p* = 0.32). In the sensitivity analyses that treated disinhibited eating as continuous variable, the results supported an interaction between health difference and disinhibited eating in predicting reaction time (*p* for interaction = 0.01) and an interaction between taste difference and disinhibited eating in predicting reaction time (*p* for interaction = 0.06).

**FIGURE 2:**
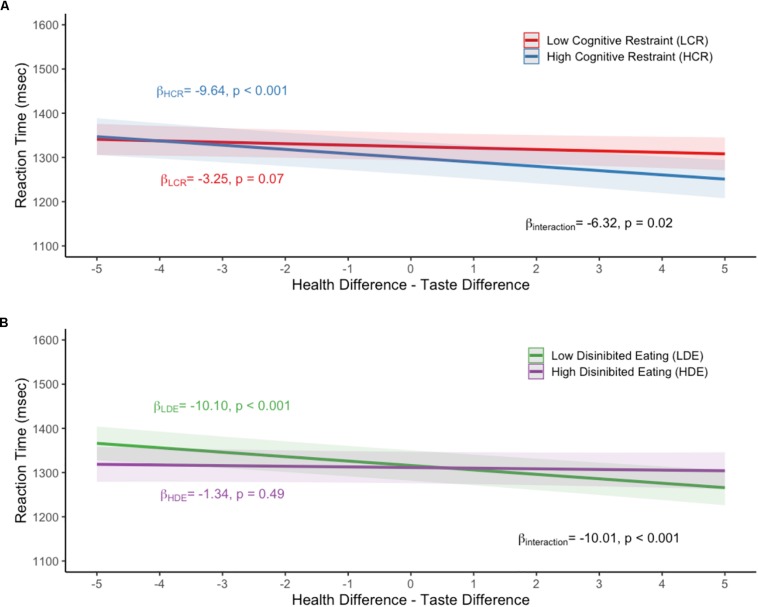
Mean reaction time values for the **(A)** median dietary cognitive restraint by health-taste difference interaction, **(B)** median disinhibited eating by health-taste difference interaction. All models adjusted for age, sex, household income, and BMI. LCR, low dietary cognitive restraint; HCR, high dietary cognitive restraint; LDE, low disinhibited eating; HDE, high disinhibited eating.

Health-taste difference results stratified by median dietary cognitive restraint and median disinhibited eating are displayed in Figure 2. A statistically significant interaction between health – taste difference and median dietary cognitive restraint (*p* for interaction = 0.02) was observed (Figure 2A). Specifically, individuals with dietary cognitive restraint scores above the median indicated preference faster (β = −9.64, *p* < 0.001) compared to those below the median (β = −3.25, *p* = 0.07) when the food item preferred was rated as healthier than tastier. A statistically significant interaction between health – taste difference and median disinhibited eating scores (*p* for interaction < 0.001) was also observed (Figure 2B). Specifically, individuals with disinhibited eating scores below the median indicated preference faster (β = −10.10, *p* < 0.001) compared to those above the median (β = −1.34, *p* = 0.49) when the food item preferred was rated as healthier than tastier. In the sensitivity analysis that treated dietary cognitive restraint and disinhibited eating as a continuous variable, statistically significant interactions were still observed (β = −0.13, *p* = 0.03; β = 0.18, *p* = 0.003, respectively).

## Discussion

In the present work, we assessed the relationship among cognitive restraint, disinhibited eating and outcomes from the dietary decision-making task including a detailed analysis of reaction times. Furthermore, we interrogated whether levels of dietary cognitive restraint and disinhibited eating were related to participants reaction times. Our results supported our hypothesis that dietary cognitive restraint was positively related to a participants’ health sensitivity and the proportion of healthy options preferred and further demonstrated a negative relationship with taste sensitivity and the proportion of tasty options preferred. It also supported our hypothesis that disinhibited eating was negatively related to a participants’ health sensitivity and the proportion of healthy options preferred and positively with taste sensitivity and the proportion of tasty options preferred. In general, our results showed that those with high cognitive restraint tend to be more sensitive to health perceptions whereas those with high disinhibited eating tend to be more sensitive to taste perceptions.

Overall, these findings are similar to self-report results previously reported by Tepper et al. (1997), showing that men with higher dietary cognitive restraint self-reported higher consumption of “healthy” foods. We found a similar relationship, that the health and taste sensitivity scores were associated with dietary cognitive restraint, using our dietary decision-making task. This provides construct validity to the use of such a task to quickly measure hypothetical food choices. Our findings also suggest that those with higher levels of dietary cognitive restraint place more emphasis on health perceptions and less on taste perceptions in their decision-making process. We do note that the opposite relationship was observed with disinhibited eating for health sensitivity and the proportion of healthy options preferred. However, this relationship was no longer statistically significant when including dietary cognitive restraint and other associated covariates. This supports the notion that dietary cognitive restraint and disinhibited eating are distinct constructs and appear to be opposing, at least within the bounds of indicating food preferences (Stunkard and Messick, 1985).

Levels of dietary cognitive restraint and disinhibited eating did not have a direct overall relationship to reaction time. In contrast to our hypotheses regarding reaction times we found that those with higher levels of dietary cognitive restraint indicated their food preference faster if the preferred food was healthier or if the preferred food was rated higher in health compared to taste. This finding suggests that to some extent those with higher levels of dietary cognitive restraint experience less conflict when making healthier food choices similar to results previously reported in women consciously attempting to lose weight (Gillebaart et al., 2016). It is possible that individuals who are actively trying to control their diet may have trained themselves to rapidly indicate a preference for items that are more obviously healthy, although this was not tested in the current experiment. There is some nuance to this finding however, as those with obesity, in our sample, tended to report higher levels of dietary cognitive restraint on average. Some previous studies have also observed an association between higher levels of dietary cognitive restraint and increased BMI (e.g., Anglé et al., 2009; Westenhoefer et al., 2013; Löffler et al., 2015; Price et al., 2015) and our results add to these. It is possible that those with increased BMI are more concerned with their dietary intake and are actively trying to control or reduce their weight through diet. It has previously been argued that dietary cognitive restraint is a measure of intent to diet, not the execution of the actual change in behavior (Lowe, 1993). Furthermore, these individuals may be executing a high level of restraint but not to the level required in order to induce weight loss or overcome other environmental influences over intake (Lowe and Levine, 2005). In line with this we found support for our hypothesis that those with higher levels of disinhibited eating had faster reaction times when they indicated preference for a food that was tastier or when the preferred food had higher taste than health ratings.

Our health-taste difference analysis corroborates our initial reaction time hypotheses. Those with high dietary cognitive restraint tended to be quicker when the preferred food was rated higher in health than taste. Conversely those with higher levels of disinhibition were quicker when the preferred food was rated higher in taste than health. These findings are consistent with previous research showing a person with a long-term goal to limit food intake will seek out and select foods that are consistent with that goal (Fishbach et al., 2003). Consequently, compared to a non-cognitively-restrained person, a cognitively restrained person may be more motivated to ensure that a food they are selecting is healthy, resulting in a faster reaction time. Conversely a person who is disinhibited shows the opposite pattern. These findings are consistent with the positive relationship between the proportion of healthy options preferred and dietary cognitive restraint and the negative relationship with disinhibited eating in our adjusted regression models.

In general, when participants indicated preference for a food during the dietary decision-making task, they did so more quickly if the health difference between the two foods was greater. This difference in speed was approximately 122 ms (∼9% difference) on average when comparing a very healthy food to a very unhealthy food. A similar association was observed with the difference in taste with participants indicating preference more quickly if the taste difference between the two foods was greater. This difference in speed was approximately 93 ms (∼7% difference) when comparing a very tasty food to a very un-tasty food. The magnitude of these values is similar to those reported by previous studies evaluating reaction time and food choice (Sullivan et al., 2014). This does speak the difficulty in making food related decisions particularly if the food is not distinctly perceived as tasty or healthy. However, those with higher dietary cognitive restraint indicated their preference quicker than those with low dietary cognitive restraint when the preferred food was healthier than the alternative. Overall, the reaction time findings suggest that those with higher dietary cognitive restraint appear to be more sensitive to health rather than taste perceptions. Conversely those with higher levels of disinhibition appear to be more sensitive to taste rather than health perceptions. It may be important to consider this heterogeneity when examining difference in food decision-making processes.

Broadly, our results suggest that metrics obtained from the dietary decision-making task are reflective of previously observed relationships between cognitive restraint and self-reported eating behaviors. Therefore, this study provides construct validity evidence that dietary decision-making tasks may offer a nuanced way to measure eating behaviors and preferences in real time. Our findings provide evidence that food preferences during a dietary decision-making task are associated with dietary cognitive restraint and disinhibited eating. However, several limitations should be noted. First participants in this study were not instructed to respond as quickly as possible and therefore were able to view the stimuli until their response was made (up to a maximum of 4 s). This limits the interpretation of reaction times as an implicit measure in our task. Reaction times within the self-regulation and food choice framework are typically defined as an implicit cognitive measure that are largely driven by non-conscious processes (Eschenbeck et al., 2016). However, for reaction times to be considered a predominately implicit measure, stimuli-presentation time should be restricted, and participants should be instructed to respond as quickly as possible to limit higher-order processing (Brown et al., 2002). Therefore, we cannot rule out that part of our reaction time measurements was due to conscious phenomena. Although we removed trials with exceptionally long reaction times in our quality control procedure, average reaction times were approximately 1.3 s. Note that reaction times of this length are similar to those observed in previous studies that have investigated food choice and reaction times that report average reaction times of approximately 1.5 s (van der Laan et al., 2014). Another limitation is that all data, except for the dietary decision-making task, were self-reported through online questionnaires. Therefore, it is likely that there is measurement error in the reporting of heights and weights, and therefore calculated BMI. Another limitation is that hunger levels were not controlled for during data collection, nor did we assess hunger level to control for it statistically. As hunger may influence food choice it is possible that hunger level differences explain some of the results. Future research should attempt to control and account for this behaviorally and/or statistically. While several aspects of the online nature of the questionnaires are problematic (such as those described above), we do highlight that it is possible that it reduced social desirability bias during the dietary decision-making task. Furthermore, the use of the online questionnaire allowed for a relatively large sample which allowed us to simultaneously control for relevant covariates. Another strength of this study is that we were able to test both behavioral (TFEQ-R18) and experimental (reaction times) variables in relation to food preference in the same sample.

Future studies are needed to assess whether the relationships observed in this study relate to actual real-world decision making and eating behavior. Decisions made when a participant will not actually consume the items may differ from decisions made when participants will actually consume the foods following selection. Future studies may also want to consider using dietary decision-making tasks to track changes in cognitive restraint to food rather than more expensive paradigms that have been used to track changes such as functional magnetic resonance imaging studies (e.g., Chen et al., 2018). Additionally, it may be useful to modify the dietary decision paradigm to train responses to specific foods or sets of foods or to have participants complete the paradigm under varying sets of instructions (i.e., thinking of health or thinking of taste). This would allow researchers to target specific eating behaviors, constructs, or food product categories as needed for specific research questions.

In summary, we found dietary cognitive restraint to be positively associated with individuals’ health sensitivity and the proportion of healthy food options preferred and negatively associated with individuals’ taste sensitivity in the dietary decision-making task. Our reaction time analysis corroborated these results indicating an implicit bias toward health perceptions for those with high levels of cognitive restraint. We show that dietary decision-making tasks are able to capture important aspects of eating behavior such as restraint and disinhibition. Therefore, this task may be useful in future studies attempting to measure and/or alter levels of dietary restraint and disinhibition in real time or under specific food comparison conditions.

## Data Availability Statement

The datasets generated for this study are available at https://github.com/IanEisenberg/Self_Regulation_Ontology/tree/master/Data.

## Ethics Statement

The studies involving human participants were reviewed and approved by the Dartmouth’s Committee for the Protection for Human Subjects and Stanford’s Institutional Review Board. The patients/participants provided their written informed consent to participate in this study.

## Author Contributions

LM, ML, and IE contributed to the conception and design of the study. IE organized the original database. TM, JB, JE, and DG-D performed the statistical analysis and interpreted the results. TM and JB wrote the first draft of the manuscript. DG-D, SM, and IE contributed to specific sections of the manuscript. All authors contributed to manuscript revision, and read and approved the submitted version.

## Conflict of Interest

The authors declare that the research was conducted in the absence of any commercial or financial relationships that could be construed as a potential conflict of interest.
