# Supplementary material for: Relationships Among Dietary Cognitive Restraint, Food Preferences, and Reaction Times
Source: Front Psychol. 2019 Oct 9;10:2256. doi: 10.3389/fpsyg.2019.02256 (PMC6794363; doi:10.3389/fpsyg.2019.02256)
Supplement: Supplementary file 1 [file Table_1.DOCX]

**Supplementary Table 1.** Foods included in the dietary decision-making task

| **Food Item** |
| --- |
| Strawberries |
| Famous Amos Cookies |
| Keebler Fudge Stripe Cookies |
| Raisins |
| Crackers |
| Red Grapes |
| Broccoli and Cauliflower |
| Chips Ahoy Cookies |
| Diet Bar |
| Cookies in Cream Chocolate Bar |
| Rice Krispy Treat |
| Reese’s Peanut Butter Cup |
| Frozen Yogurt |
| 100 Grand |
| Flaming Cheetos |
| Cookies |
| Doritos Ranch Chips |
| Raspberry Sorbet |
| Ruffles Chips |
| White Grapes |
| Wheat Crisps |
| Butterfinger |
| Oreos |
| Celery |
| Lays Classic Chips |
| KitKat |
| Carrots |
| Hostess HoHos |
| Granny Smith Apples |
| Toblerone |
| Red Delicious Apples |
| Brownie |
| Orange Jell-O |
| Banana |
| Strussel |
| Slimfast Chocolate |
| Keebler Rainbow Cookies |
| Orange |
| Lindt Chocolate |
| Ghiradelli |
| Ice Cream Sandwich |
| Slimfast Vanilla |
| Muffin |
| Special-K Bar |
| Mixed Berry Yogurt |
| Cherry Ice cream |
| Twix |
| Cranberries |
| Mrs Fields Cookies |
| Blueberry Yogurt |
