# Supplementary material for: Relationships Among Dietary Cognitive Restraint, Food Preferences, and Reaction Times
Source: Front Psychol. 2019 Oct 9;10:2256. doi: 10.3389/fpsyg.2019.02256 (PMC6794363; doi:10.3389/fpsyg.2019.02256)
Supplement: Supplementary file 2 [file Table_2.DOCX]

**Supplementary Table 2.**

|  | **B** | **Std. error** | **p-value** |
| --- | --- | --- | --- |
| **Cognitive Restraint** |  |  |  |
| Low | -2.91 | 1.77 | 0.09 |
| High | -9.29 | 2.05 | < 0.001 |
| Interaction | -6.59 | 2.75 | 0.017 |
| **Disinhibited eating** |  |  |  |
| Low | -9.75 | 1.84 | <0.001 |
| High | -0.09 | 1.96 | 0.609 |
| Interaction | -9.67 | 1.82 | <0.001 |

Results of the regressions predicting reaction time from health-taste differences

scores ranging from -8 to 8 using median splits for cognitive restraint and disinhibited

eating scores.
