# Supplementary material for: Relationships Among Dietary Cognitive Restraint, Food Preferences, and Reaction Times
Source: Front Psychol. 2019 Oct 9;10:2256. doi: 10.3389/fpsyg.2019.02256 (PMC6794363; doi:10.3389/fpsyg.2019.02256)
Supplement: Supplementary file 3 [file Table_3.DOCX]

**Supplementary Table 3.** Correlations between variables of interest and demographic variables

|  | TS | HS | PH | PT | CR | UE | EE | DE | Sex | Age | Race | BMI | HE | HI | RT |
| --- | --- | --- | --- | --- | --- | --- | --- | --- | --- | --- | --- | --- | --- | --- | --- |
| Taste Sensitivity (TS) | 1 |  |  |  |  |  |  |  |  |  |  |  |  |  |  |
| Health Sensitivity (HS) | **-0.18**** | 1 |  |  |  |  |  |  |  |  |  |  |  |  |  |
| Proportion Healthy (PH) | 0.02 | **0.46**** | 1 |  |  |  |  |  |  |  |  |  |  |  |  |
| Proportion Tasty (PT) | **0.56**** | **-0.14*** | **-0.18*** | 1 |  |  |  |  |  |  |  |  |  |  |  |
| Cognitive Restraint (CR) | **-0.11*** | **0.16**** | **0.15**** | **-0.12*** | 1 |  |  |  |  |  |  |  |  |  |  |
| Uncontrolled Eating (UE) | -0.03 | **-0.14*** | -0.08 | -0.06 | **-0.09*** | 1 |  |  |  |  |  |  |  |  |  |
| Emotional Eating (EE) | -0.03 | **-0.10*** | -0.08 | -0.06 | -0.02 | **0.69**** | 1 |  |  |  |  |  |  |  |  |
| Disinhibited Eating (DE) | -0.03 | **-0.13*** | **-0.09*** | -0.07 | -0.05 | **0.88**** | **0.95**** | 1 |  |  |  |  |  |  |  |
| Sex | **0.14*** | -0.01 | 0.06 | 0.08 | **0.10*** | -0.05 | **0.11*** | 0.05 | 1 |  |  |  |  |  |  |
| Age | **0.09*** | 0.01 | 0.00 | 0.01 | **0.10*** | **-0.14*** | 0.00 | -0.06 | **0.16**** | 1 |  |  |  |  |  |
| Race | 0.04 | 0.00 | **-0.10*** | **0.08** | -0.02 | 0.00 | -0.05 | -0.03 | 0.00 | 0.08 | 1 |  |  |  |  |
| BMI | -0.04 | **-0.14*** | -0.05 | -0.01 | 0.01 | **0.29**** | **0.38**** | **0.37**** | 0.05 | 0.07 | -0.03 | 1 |  |  |  |
| Highest Education (HE) | -0.04 | 0.05 | -0.07 | 0.03 | 0.07 | 0.02 | 0.00 | 0.01 | **-0.09*** | **0.09*** | -0.07 | **-0.11*** | 1 |  |  |
| Household Income (HI) | 0.02 | **0.10*** | -0.02 | 0.02 | **0.16** | -0.04 | 0.00 | -0.02 | 0.08 | **0.14*** | -0.01 | -0.04 | **0.26**** | 1 |  |
| Reaction Time (RT) | **0.31**** | -0.05 | -0.07 | **0.29**** | 0.03 | -0.07 | 0.01 | -0.02 | **0.09*** | **0.27**** | 0.02 | -0.05 | 0.06 | -0.03 | 1 |

All reported values are Pearson’s r
* indicates p <0.05; ** indicates p<0.001
